# Ionic Liquids and Deep Eutectic Solvents for Polyphenol Extraction: Opportunities and Limitations

**DOI:** 10.3390/ijms27083538

**Published:** 2026-04-15

**Authors:** Gonçalo P. Rosa, Maria Carmo Barreto, Ana M. L. Seca, Diana C. G. A. Pinto

**Affiliations:** 1 University of the Azores, Faculty of Sciences and Technology, Centre for Ecology, Evolution and Environmental Changes (cE3c), Azorean Biodiversity Group & Global Change and Sustainability Institute (CHANGE), 9500-321 Ponta Delgada, Portugal; goncalo.p.rosa@uac.pt (G.P.R.); maria.cr.barreto@uac.pt (M.C.B.); 2 LAQV-REQUIMTE, Department of Chemistry, University of Aveiro, 3810-193 Aveiro, Portugal; diana@ua.pt

**Keywords:** polyphenols, ionic liquids, deep eutectic solvents, sustainability, flavonoids, tannins

## Abstract

Polyphenols are structurally diverse plant secondary metabolites with broad biological activities and growing applications across the food, health, and materials sectors. Conventional extraction based on organic solvents (e.g., methanol, ethanol) is often energy-intensive, inefficient, and environmentally burdensome. Ionic liquids (ILs) and deep eutectic solvents (DESs) have therefore emerged as greener alternatives for polyphenol extraction. This review evaluates recent advances in solvent design, extraction performance, and process sustainability. Imidazolium-based ILs frequently achieve high yields and selectivity, particularly when coupled with ultrasound or microwave-assisted extraction, but high cost, synthetic complexity, viscosity-related constraints, and potential toxicity hinder scaleup. By contrast, DESs—especially those derived from choline chloride or lactic acid—are easier to prepare, less costly, and more compatible with industrial implementation, with efficiency enhanced by tailoring hydrogen bond networks, water content, and process intensification. Critical downstream challenges persist for both solvent classes, notably in extract purification and solvent recovery due to low volatility; approaches such as resin adsorption, antisolvent precipitation, and direct formulation have been explored. Overall, ILs and DESs represent compelling alternatives to conventional solvents, and future progress will depend on integrated extraction–recovery strategies, systematic solvent selection, and validation under scalable, sustainable processing conditions.

## 1. Introduction

**Figure 1:**
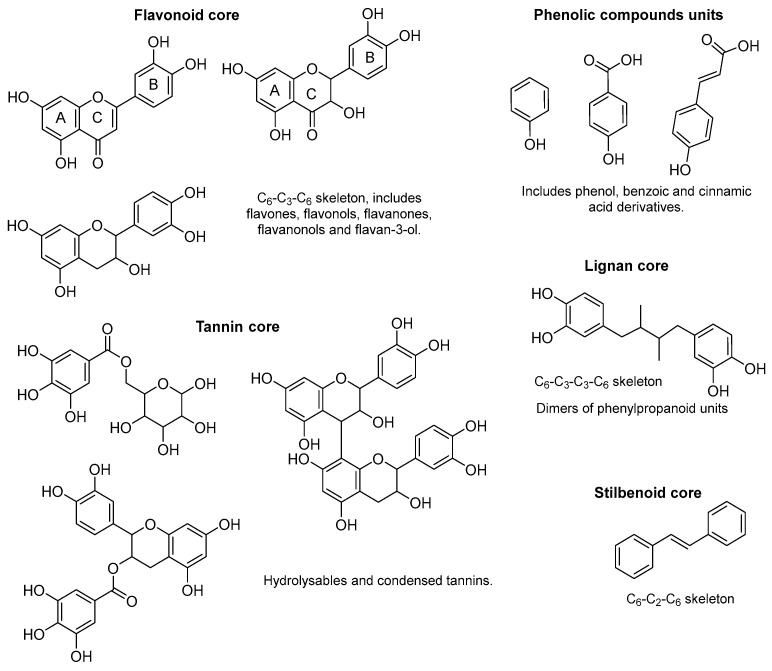
Representative structural frameworks of major polyphenol classes.

Polyphenols constitute one of the most abundant and structurally diverse classes (Figure 1) of secondary metabolites in plants, playing crucial roles in plant defense mechanisms and contributing significantly to the organoleptic and nutritional properties of fruits, vegetables, and derived products [1].

From a biomedical perspective, polyphenols are widely associated with antioxidant and anti-inflammatory activities, including the modulation of reactive oxygen and nitrogen species as well as the regulation of endogenous antioxidant defenses [2]. Moreover, numerous studies have explored their potential roles in a broad spectrum of chronic conditions, such as metabolic and cardiovascular disorders, cancer, osteoporosis, and neurodegenerative diseases [3,4,5,6,7,8,9]. Beyond health-related applications, polyphenols have also gained relevance as functional ingredients across several industrial sectors, ranging from food colorants and packaging materials to cosmetics, coatings, and polymer-based products [10,11]. This wide applicability has driven sustained research and development focused on polyphenol rich extracts and purified compounds [12,13,14].

Conventional extraction techniques typically rely on organic solvents such as methanol, ethanol, acetone, or their aqueous mixtures. Although effective, these approaches present several well-recognized limitations, including solvent toxicity, thermal degradation of target compounds, environmental impact, and limited selectivity toward specific polyphenolic subclasses. While ionic liquids and deep eutectic solvents have been proposed as alternative extraction media, they do not inherently overcome all these limitations. In particular, thermal degradation remains primarily governed by the extraction technique and operating conditions, and selectivity toward specific polyphenolic subclasses is only partially improved, depending on the solvent system and matrix interactions [15,16,17,18].

The growing demand for high-quality polyphenolic ingredients has intensified the need for extraction strategies capable of preserving their structural integrity and biological activity. At the same time, increasing adherence to green chemistry principles has highlighted the importance of developing more sustainable extraction systems that balance efficiency with environmental and safety considerations. However, the classification of these systems as “green” requires careful consideration and, ideally, a full life cycle assessment (LCA), which is still rarely performed.

In response, alternative methods—such as ultrasound-assisted extraction, microwave-assisted extraction, and supercritical fluid extraction—have been introduced to enhance mass transfer and reduce processing times [17,18,19].

Within this context, ILs and DESs have emerged as alternative media for polyphenol extraction [20,21,22,23]. Many of these systems exhibit low volatility, which is often considered advantageous compared to conventional organic solvents; however, this property is strongly dependent on their specific chemical composition, and exceptions have been reported [24]. Both ILs and DESs comprise chemically diverse families, encompassing a wide range of structures and physicochemical properties. Their tunable characteristics and ability to establish hydrogen-bonding and electrostatic interactions with target molecules can contribute to enhanced solvation and, in some cases, improved extraction performance. Nevertheless, given the structural diversity of ILs and DES, these effects are highly system-dependent, and broad generalizations regarding their behavior or sustainability should be avoided without case-by-case evaluation.

Accordingly, the present review examines recent advances in the use of ILs and DESs for polyphenol extraction, with particular focus on solvent design, interaction mechanisms, recent developments, existing limitations, and realistic opportunities for improving sustainability and enabling larger scale implementation. By integrating these aspects, this work aims to provide a clearer understanding of both the potential and the constraints associated with these emerging solvents as alternative extraction media within green chemistry frameworks.

## 2. Methodology of the Literature Review

### 2.1. Search Strategy and Sources

A structured literature survey was performed to identify studies addressing the use of ILs and DESs as extraction media for polyphenols from plant-derived matrices and agro-industrial by-products. Searches were conducted using major scientific indexing databases (e.g., Scopus, Web of Science, and PubMed), complemented by manual screening of reference lists from key papers and relevant reviews identified during the search process.

### 2.2. Keywords and Query Structure

Search queries combined solvent-related and target-related terms, including: “polyphenol*”, “phenolic compound*”, “flavonoid*”, “anthocyanin*”, “tannin*” with “ionic liquid*”, “deep eutectic solvent*”, “natural deep eutectic solvent*”, “NaDES”, as well as process terms such as “extraction”, “ultrasound-assisted extraction”, “microwave-assisted extraction”, and “aqueous biphasic system”.

### 2.3. Time Frame and Topical Scope

The scope focused on literature that reflects contemporary extraction practice and solvent development, prioritizing studies published from 2010 to 2025, while including seminal earlier contributions when required for conceptual completeness. The review targeted works in which ILs or DESs were explicitly used as extraction media (neat or aqueous mixtures), including processes coupled to intensification (e.g., ultrasound, microwave) and/or integrated downstream steps (e.g., purification, solvent recovery).

### 2.4. Inclusion Criteria

For inclusion in this review, studies were required to report the extraction of polyphenols, either as total phenolic content and/or as identified individual polyphenolic compounds, using ILs or DESs. In addition, the studies had to provide sufficient experimental detail to allow interpretation of the solvent composition and extraction conditions, including at least the solvent system used, the solid–liquid ratio, and the extraction time and temperature or applied energy input. Finally, only studies presenting quantitative extraction outcomes—such as yields, recoveries, total phenolic contents, or compound concentrations—and/or a comparative assessment of performance relative to conventional solvents or extraction techniques were considered eligible.

### 2.5. Exclusion Criteria

Studies were excluded from the analysis when ILs or DESs were employed solely as analytical additives rather than as extraction media. Exclusion also applied to studies focusing on non-phenolic target compounds without reporting any outcomes related to polyphenols. In addition, studies that did not present quantitative extraction results or failed to describe solvent composition and extraction conditions with sufficient clarity to allow meaningful comparison were not considered.

### 2.6. Data Extraction and Synthesis Approach

For each eligible study, key parameters were extracted: solvent composition (cation/anion for ILs; hydrogen bond acceptor to hydrogen bond donor ratio (HBA/HBD) and molar ratio for DESs), water content (when applicable), extraction technique and operating conditions, matrix type, and reported polyphenol outcomes. The narrative synthesis emphasizes: (i) solvent-structure/property considerations linked to performance, (ii) the role of process intensification, and (iii) downstream constraints (purification and solvent recovery), as these are determinant factors for translation beyond laboratory demonstrations.

## 3. Ionic Liquids

### 3.1. Structural Features and Solvent–Solute Interactions

Ionic liquids (ILs) are salts composed of organic cations and organic or inorganic anions, typically characterized by melting points below 100 °C and thus often liquid at or near room temperature [25]. Although this definition is widely accepted and appropriate for conventional ILs, the emergence of task-specific ionic liquids has introduced additional functional and compositional complexity that is not fully captured by a purely structural definition.

**Figure 2:**
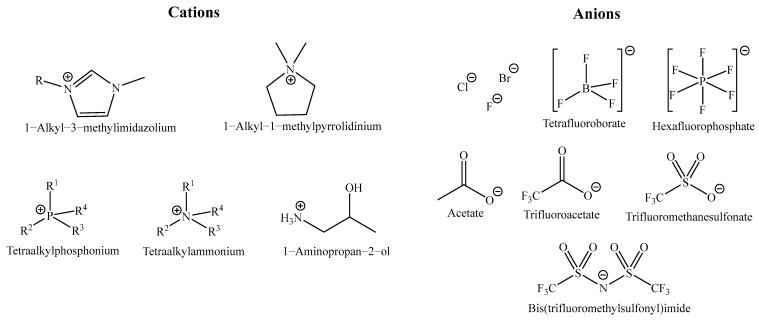
Most common cations and anions used for IL preparation.

Their early adoption as alternative extraction media was driven by their negligible vapor pressure, high thermal stability, and, most importantly, the possibility of tailoring solvent properties through rational ion-pair design [26,27]. The most employed cation–anion combinations reported in extraction studies are depicted in Figure 2.

In polyphenol extraction, IL performance is governed primarily by specific solvent–solute interactions rather than by bulk polarity alone. Hydrogen bonding between anions and phenolic hydroxyl groups, π–π stacking interactions between aromatic cations (particularly imidazolium-based systems) and phenolic rings, and electrostatic contributions collectively determine solubilization efficiency and selectivity [25,28]. These interactions are highly sensitive to cation structure (aromaticity, alkyl chain length, functionalization) and anion basicity or coordinating ability, resulting in pronounced differences in extraction behavior across IL families.

An additional parameter that may influence extraction performance is the ionicity, or degree of ion dissociation, of ionic liquids. This property affects the effective ionic strength of the medium and can modulate solute–solvent interactions. In systems with high ionic strength, the extraction of labile compounds such as polyphenols may be hindered due to possible degradation, complexation, or reduced solubility, depending on the specific IL composition and operating conditions [29].

**Figure 3:**
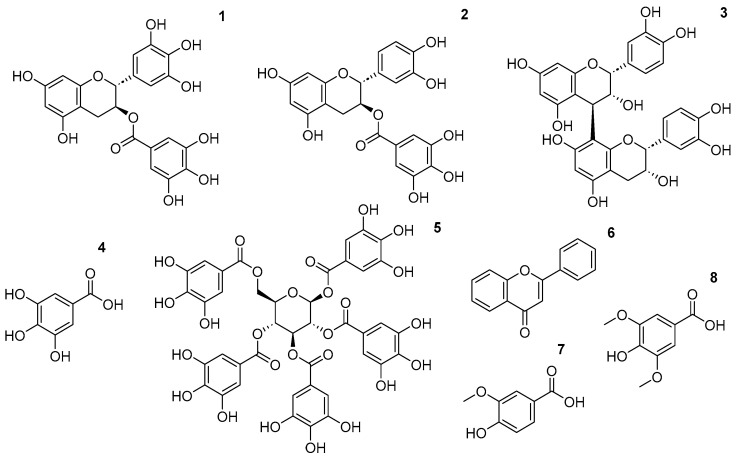
Main polyphenol extracted with ILs.

This molecular tunability enables the design of task-specific ILs, tailored to favor particular polyphenol subclasses (Figure 3).

However, it also introduces complexity, as optimal solvent performance becomes strongly dependent on the chemical nature of both the target compounds and the biomass matrix. The diversity of IL systems explored for polyphenol extraction, together with their targets, extraction techniques, and reported limitations, is summarized in Table 1.

### 3.2. Ionic Liquids in Polyphenol Extraction: Performance Trends

As illustrated in Table 1, imidazolium-based ILs dominate the literature on IL-assisted polyphenol extraction [29,30,31,32,33]. These systems have been applied to a wide range of plant matrices and frequently achieve extraction efficiencies comparable to or exceeding those obtained with conventional organic solvents. Particularly strong performance has been reported for flavonoids, catechins, tannins, and phenolic acids, especially in matrices where conventional solvents exhibit limited selectivity.

Importantly, ILs do not consistently provide a universal increase in total phenolic content. Instead, many studies report selective enrichment of specific phenolic fractions, reflecting the dominant role of solvent–solute interactions over nonspecific solvation effects [33,37]. Anion identity emerges as a decisive factor, with hydrogen-bond-accepting or weakly coordinating anions often enhancing phenolic recovery [29,37]. This selectivity distinguishes IL-based extraction from traditional solvent systems but also complicates direct comparison across studies and matrices.

These observations reinforce the notion that ILs function best as precision tools rather than as broadly applicable extraction solvents. Consequently, their performance must be interpreted within the specific chemical and structural context of the biomass under investigation, rather than extrapolated across different systems. The limitations summarized in Table 1 reflect both reported constraints and those inferred from the analysis of the extraction systems and, therefore represent a critical evaluation rather than exclusively experimental observations.

### 3.3. Process Intensification and Operational Constraints

The combination of ILs with process intensification techniques has proven central to their reported success in polyphenol extraction. Ultrasound-assisted extraction (UAE) and microwave-assisted extraction (MAE) are particularly effective, as they enhance mass transfer, promote cell wall disruption, and reduce extraction times when ILs are used as solvents [30,31,32]. More complex configurations, including ultrasound–microwave-assisted extraction (UMAE) and aqueous biphasic systems incorporating ILs, further demonstrate the versatility of these solvents beyond simple solid–liquid extraction [31,38]. In these systems, ILs may function simultaneously as extraction media and phase-forming or separation-enhancing agents. As summarized in Table 1, such intensified approaches often reduce extraction times from hours to minutes while maintaining or improving extraction efficiency.

Nevertheless, intensified processing can also exacerbate certain limitations of ILs, such as viscosity-related mass transfer constraints or difficulties in solvent recovery, underscoring the importance of balancing extraction performance against downstream feasibility.

### 3.4. Intrinsic Limitations, Toxicity, and Sustainability Constraints

Despite their demonstrated technical potential, ILs face substantial barriers to large-scale implementation. High production costs, complex synthesis routes, and concerns regarding toxicity and environmental persistence remain the most frequently cited limitations [39]. These issues are particularly relevant for applications targeting food, cosmetics, or pharmaceutical sectors, where regulatory acceptance is critical.

In addition, the high viscosity of many ILs can hinder mass transfer, complicated solvent handling, and pose challenges for downstream processing and solvent recycling.

In fact, as summarized in Table 1, information regarding solvent recovery and recyclability is only sporadically reported in IL-based extraction studies. In some cases, efficient recovery strategies are described, such as magnetic separation in magnetic ionic liquids or phase reuse in phosphonium-based systems. However, these examples remain limited, and systematic evaluation of solvent reuse is still lacking across most studies. Another recurring issue is the reliance on empirical screening approaches to identify suitable ILs, which are both time- and resource-intensive and undermines claims of sustainability [40]. Although recent spectroscopic and predictive methodologies have been proposed to rationalize solvent selection and reduce unnecessary experimentation [33,40], their adoption remains limited.

The collective limitations reported across the studies summarized in Table 1 highlight why, despite promising extraction efficiencies and selectivity, ILs have not achieved widespread industrial usage. These constraints also help explain the growing shift in research focus toward DESs, which aim to retain many of the advantageous interaction features of ILs while offering improved economic and environmental profiles.

In parallel with these challenges, increasing attention has been devoted to the development of more sustainable ionic liquids, including bio-based and task-specific systems designed to reduce toxicity and improve biodegradability [41]. These advances include ionic liquids derived from naturally occurring compounds, such as amino acids, organic acids, and cholinium-based systems, as well as the design of more benign cation–anion combinations [42]. Nevertheless, despite these developments, within the specific context of polyphenol extraction, the available literature remains predominantly focused on conventional ionic liquids, particularly imidazolium-based systems, which continue to be the most extensively studied. This imbalance further reinforces the current interest in alternative solvent systems such as DESs. Consequently, while acknowledging the growing relevance of greener ionic liquids, the present review primarily addresses those systems for which sufficient data on polyphenol extraction is currently available. Further studies exploring these emerging ionic liquids in polyphenol extraction are needed to determine whether they can effectively overcome the limitations associated with conventional systems.

## 4. Deep Eutectic Solvents

### 4.1. Definition and General Characteristics

Deep eutectic solvents (DESs) are liquid systems formed by mixing an HBA and an HBD. They interact predominantly through hydrogen bonding to generate a eutectic mixture with a melting point significantly lower than those of the individual components [43,44]. Unlike ILs, DESs are typically prepared by simple heating and mixing, without the need for complex synthesis or purification steps, which contributes to their lower cost and improved accessibility.

**Figure 4:**
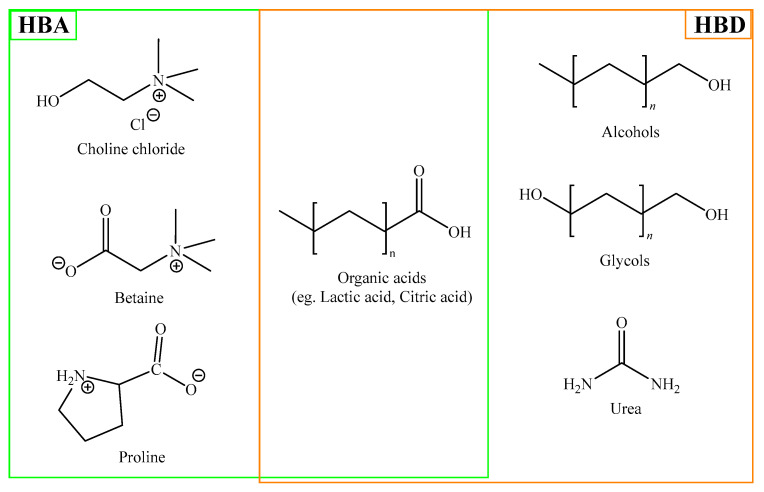
Molecules commonly used as HBA and HBD for DES preparation.

These systems share several physicochemical features with ILs, including generally low volatility, non-flammability, and high solvation capacity for a wide range of organic and inorganic compounds [45]. However, a key distinguishing factor lies in their compositional variability: DES properties can be adjusted through the selection and molar ratio of HBA and HBD components, many of which are naturally occurring or biocompatible molecules. Nevertheless, their physicochemical behavior is strongly dependent on composition, and the addition of water or other components may significantly alter the hydrogen-bond network that defines the DES structure. In such cases, the extent to which DES-specific interactions are preserved becomes system-dependent, and the resulting medium may progressively resemble a conventional solution. The most used HBAs and HBDs reported in DES preparation are shown in Figure 4.

Deep eutectic solvents can be categorized into four major groups: Type I (organic salt +metal), Type II (organic salt +metal salt hydrate), Type III (organic acids +HBD), and Type IV (aluminum/zinc chloride +HBD). Within Type III, there are natural DESs (NaDESs), which are formed from a mixture of two or three natural molecules (e.g., organic acids, sugars, and amino acids), being the most widely applied in biomass processing and polyphenol extraction [46].

From a mechanistic perspective, DES efficiency in polyphenol extraction arises primarily from extensive hydrogen-bond networks, which can simultaneously solubilize phenolic compounds and disrupt plant cell wall structures. These effects are strongly modulated by viscosity, polarity, acidity, and water content, parameters that must be carefully balanced to maximize extraction performance.

### 4.2. DES in Polyphenol Extraction: General Performance Trends

Compared with ILs, DESs have been investigated across a broader diversity of plant matrices and agro-industrial by-products, reflecting their lower cost, simpler preparation, and more favorable environmental profile. Numerous studies report extraction efficiencies equal to or higher than those achieved with conventional organic solvents, particularly for total phenolic content and flavonoid-rich extracts [47,48,49,50,51,52,53,54,55].

Importantly, DESs frequently outperform conventional solvents not solely by increasing solubility but by enhancing matrix disruption. Several works report substantial alteration of plant tissue morphology following DES extraction, consistent with partial dissolution of lignocellulosic components and improved accessibility of intracellular polyphenols [49,56]. This dual role—acting as both solvent and matrix-disrupting agent—distinguishes DESs from most conventional extraction media.

As with ILs, DESs do not exhibit universal behavior. Extraction outcomes depend strongly on solvent composition, molar ratio, water content, and extraction conditions, reinforcing the importance of solvent design tailored to the target polyphenol class and biomass.

### 4.3. Choline Chloride-Based DES

Choline chloride (ChCl)-based DES clearly constitute the most extensively investigated class of DESs for polyphenol extraction, as evidenced by the breadth of solvent compositions, target compounds, and biomass matrices compiled in Table 2. This predominance arises from a unique combination of factors, including the low cost and wide availability of choline chloride, its favorable toxicological profile, regulatory acceptance for food- and cosmetic-related applications, and its strong hydrogen bond accepting ability, which is central to the solubilization of phenolic compounds [43,47].

**Figure 5:**
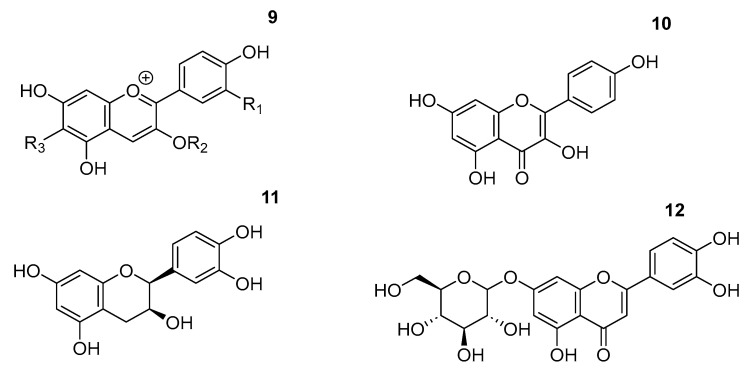
Specific polyphenols extracted with choline-chloride-based DES.

When paired with appropriate hydrogen bond donors—most notably organic acids, polyols, or multicomponent mixtures—ChCl-based DES frequently outperform conventional solvents such as methanol or aqueous ethanol, not only in terms of total extraction yield but also with respect to selectivity (Figure 5; Table 2).

Acidic ChCl-based DESs tend to favor the extraction of phenolic acids and anthocyanins, reflecting the combined effects of medium acidity, extensive hydrogen-bonding networks, and possible protonation of functional groups within the plant matrix. In contrast, polyol-based ChCl DESs consistently show improved performance for flavonoids, especially glycosylated flavonoids, likely due to a more balanced combination of polarity, hydrogen bonding capacity, and reduced chemical aggressiveness.

A comparative examination of the studies summarized in Table 2 further indicates that the performance of ChCl-based DESs is strongly dependent on the nature of the plant matrix and the targeted polyphenol class. Lignocellulosic or structurally recalcitrant matrices tend to benefit more from acidic DESs capable of partially disrupting cell wall components, whereas less recalcitrant matrices often respond favorably to less acidic, viscosity-controlled systems, reinforcing the view that ChCl-based DESs should be regarded as task-specific solvents rather than universally applicable extraction media.

**Figure 6:**
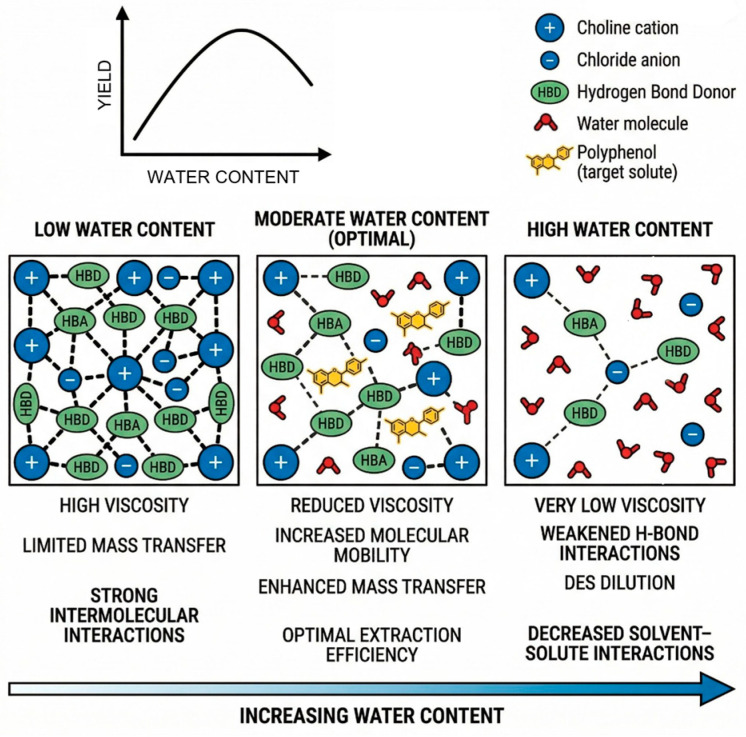
Schematic representation of the effect of water content on DES nanostructure and extraction efficiency.

Water content emerges as one of the most critical parameters governing extraction efficiency in ChCl-based DES systems. Moderate water addition effectively reduces solvent viscosity, enhances mass transfer, and accelerates extraction kinetics (Table 2). However, excessive dilution disrupts the eutectic structure, weakens specific solvent–solute interactions, and can ultimately reduce extraction performance [49,58]. This effect is schematically illustrated in Figure 6. This trade-off between improved fluidity and preservation of solvent structure shows the need for careful optimization of water content rather than empirical dilution strategies.

Despite their frequently high extraction efficiencies, many ChCl-based DES systems reported in Table 2 present relevant practical challenges, including high residual viscosity, difficulties in solvent recovery, and the potential co-extraction of undesired matrix components. In addition, choline chloride-based systems may present further limitations related to handling and stability, such as characteristic odor and possible degradation under certain processing conditions, particularly at elevated temperatures. From a physicochemical perspective, these systems may also exhibit relatively high ionic strength and sensitivity to temperature and composition, factors that can influence both solvent stability and extraction performance [59]. These constraints emphasize that extraction yield alone is insufficient to evaluate solvent performance and that downstream compatibility and recyclability must be considered from the earliest stages of solvent and process design.

Taken together, the evidence summarized in Table 2 demonstrates that choline chloride-based DESs constitute a versatile platform for polyphenol extraction. However, their effectiveness relies on a delicate balance between solvent composition, water content, matrix characteristics, and the intended analytical or technological objective. This complexity underlies both the widespread success of these systems and the variability of outcomes reported across the literature.

### 4.4. Non-Choline Chloride-Based DES

**Figure 7:**
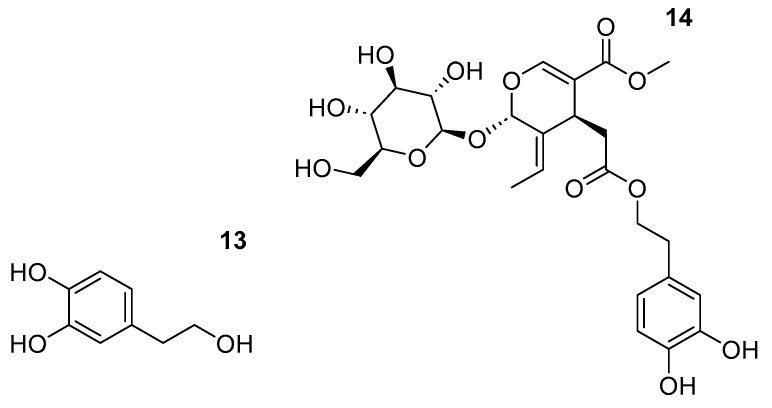
Some of the polyphenol extracted with non-ChCl-based DES.

Beyond choline chloride-based formulations, a wide range of alternative DES systems has been explored for polyphenol extraction, as summarized in Table 3. The chemical structure of specific polyphenols pointed in this table is shown in Figure 7.

Among these, lactic acid-based DESs have attracted particular attention, largely due to their strong hydrogen bond donating ability, inherent acidity, and favorable biocompatibility profile. These characteristics make lactic acid-based systems especially effective for solubilizing phenolic compounds that interact strongly with plant cell wall components or are present in tightly bound forms.

The studies compiled in Table 3 indicate that lactic acid-based DESs frequently achieve extraction yields comparable to or exceeding those obtained with conventional organic solvents, particularly for total polyphenols and flavonoid-rich extracts. Their performance is often enhanced when combined with moderate water content or assisted extraction techniques, such as ultrasound or microwave irradiation. However, the strong acidity of these systems can also introduce selectivity biases, favoring certain phenolic subclasses while potentially promoting the degradation of acid-sensitive compounds if extraction conditions are not carefully controlled.

In addition to lactic acid-based mixtures, DESs incorporating sugars, amino acids, polyols, or organic acid combinations further expand the solvent design space available for polyphenol extraction (Table 3). These systems often display lower acidity and, in some cases, reduce chemical aggressiveness toward the biomass matrix, making them suitable for extracting more labile polyphenols. Sugar- and polyol-based DESs, in particular, have been reported to perform well for flavonoids and phenolic glycosides, likely due to the formation of extensive but less disruptive hydrogen-bonding networks.

A key observation emerging from Table 3 is the pronounced system-specific behavior of non-choline chloride DESs. Unlike ChCl-based systems, which exhibit relatively consistent performance trends across matrices, alternative DESs often display narrower applicability windows, with extraction efficiency strongly dependent on precise solvent composition, molar ratios, and water content. This sensitivity underscores the importance of solvent tailoring but also limits the generalizability of results across different biomass sources.

Water content once again plays a central role in determining extraction outcomes. As reflected in Table 3, moderate hydration typically can improve solvent fluidity and mass transfer, whereas excessive water addition weakens solvent–solute interactions and diminishes extraction efficiency. In highly viscous systems, water acts as a necessary modulator of transport properties; however, it also reduces the distinct physicochemical environment that underpins DES performance.

From a practical standpoint, several non-ChCl DESs present challenges related to viscosity, solvent recovery, and reproducibility. Multicomponent systems, while offering fine-tuned solvation environments, can complicate solvent preparation, scale-up, and reuse. These issues highlight a trade-off between solvent design flexibility and operational simplicity that must be carefully considered when selecting DES systems for applied extraction workflows.

Overall, the data summarized in Table 3 demonstrate that lactic acid-based and other alternative DESs provide valuable and often highly effective options for polyphenol extraction, particularly when tailored to specific targets or matrices. However, their broader adoption will depend on improved mechanistic understanding, streamlined solvent formulations, and integration with downstream processing strategies that account for viscosity, acidity, and solvent recyclability.

### 4.5. Process Intensification and Practical Considerations

The high viscosity that characterizes many DESs represents one of the main practical constraints for their application in solid–liquid extraction. As reflected by numerous studies summarized in Table 2 and Table 3, this property can limit mass transfer, cause slow extraction kinetics, and hinder solvent penetration into complex biomass matrices when conventional extraction approaches are employed. To overcome these limitations, DES-based extraction has been extensively combined with process intensification techniques, most notably UAE and MAE [59,77,78,79,80,81,82,83,84,85,86].

Ultrasound-assisted extraction is particularly effective in DES systems, as acoustic cavitation enhances solvent penetration, disrupts plant cell structures, and compensates for the reduced diffusivity associated with high-viscosity media [59,77,78,79,80,83,86]. Across a wide range of plant matrices, UAE has been shown to significantly reduce extraction time while maintaining or improving polyphenol yields, even when relatively viscous DES formulations are employed (Table 2 and Table 3). Microwave-assisted extraction provides complementary benefits by promoting rapid volumetric heating, which decreases solvent viscosity in situ and accelerates solute desorption from the biomass [81,84,85,86]. In many cases, the combination of DESs with UAE or MAE shifts extraction times from hours to minutes without compromising extraction efficiency or selectivity.

Beyond improving extraction efficiency, process intensification also influences solvent performance in a more fundamental way. By mitigating transport limitations, intensified techniques allow the intrinsic solvent–solute interactions of DESs—such as hydrogen bonding capacity, polarity matching, and acidity effects—to dominate extraction behavior [47,49,59,69]. This effect partially explains why DESs that exhibit limited performance under conventional stirring conditions may display markedly improved extraction efficiency when coupled with ultrasound or microwave irradiation.

Despite these advantages, process intensification does not fully resolve the practical challenges associated with DES-based extraction. Table 2 and Table 3 evidence that solvent recovery and recyclability are rarely addressed in DES-based extraction studies, despite the extensive number of reports on their extraction efficiency. In most cases, studies focus primarily on yield improvement, while downstream processing and solvent reuse remain largely unexplored. Even when purification of target compounds is achieved, solvent recovery is seldom discussed, highlighting a critical gap between laboratory performance and process sustainability.

High residual viscosity and low vapor pressure remain critical issues for downstream processing, particularly for solvent recovery and recycling [87]. As discussed in Section 5, these physicochemical properties preclude solvent removal by conventional evaporation and often necessitate additional purification strategies, such as adsorption on macroporous resins or antisolvent-induced precipitation [73,77,84]. Consequently, gains achieved during extraction can be offset by increased downstream complexity if solvent recovery is not considered during process design.

At the same time, the non-volatile nature of DESs opens up alternative processing opportunities that are less accessible with conventional solvents. Several studies propose bypassing solvent removal altogether by directly incorporating DES extracts into end-use formulations, particularly in cosmetic, pharmaceutical, or nutraceutical contexts where DES components are biocompatible [47,60,64]. In such cases, viscosity and solvent retention become design parameters rather than intrinsic drawbacks, if extract stability, bioavailability, and regulatory acceptance are adequately addressed.

From a practical perspective, the studies reviewed highlight that the successful implementation of DES-based extraction requires a holistic approach, in which solvent composition, water content, extraction technique, and downstream strategy are optimized simultaneously [49,58,67]. Process intensification should therefore be viewed not as an auxiliary enhancement, but as an integral component of DES-based extraction workflows, enabling these solvents to reach their full potential while mitigating inherent physicochemical limitations.

## 5. Sample Purification and DES Recovery

Following extraction, the recovery of polyphenols from IL- and DES-based extracts represent one of the most critical bottlenecks for process feasibility and sustainability. The same physicochemical properties that underpin the effectiveness of these solvents, namely negligible vapor pressure, strong solvation capacity, and extensive hydrogen-bonding networks, simultaneously hinder solvent removal by conventional evaporation-based approaches [87,88]. Consequently, alternative strategies must be employed, including antisolvent precipitation, liquid–liquid extraction, adsorption onto solid supports, and chromatographic separation. However, these approaches often require additional solvents, multiple processing steps, or complex operational conditions, which can increase process cost and may offset the advantages observed at the extraction stage. As a result, downstream processing must be considered an integral component of smart-solvent-based extraction workflows rather than a secondary optimization step.

This challenge is particularly pronounced for DES systems, whose high viscosity and low volatility complicate phase separation and mass transfer during purification. While ILs share similar limitations, their cost and synthesis complexity can vary significantly depending on their chemical structure. Although recent developments, including bio-based ionic liquids, have contributed to reducing these constraints, cost and synthetic effort remain relevant considerations in many systems, reinforcing the importance of efficient solvent recovery strategies. Consequently, extraction yield alone is an insufficient metric for evaluating the performance of ILs and DESs unless accompanied by a realistic assessment of downstream compatibility.

### 5.1. Solid–Liquid Adsorption and Resin-Based Recovery Strategies

Among the approaches reported for polyphenol recovery from DES-based extracts, adsorption onto macroporous resins has emerged as the most widely applied and versatile strategy. Resins such as D101, X5, AB-8, and related polymeric materials exhibit high affinity for phenolic compounds, enabling their selective adsorption from viscous DES matrices followed by desorption using ethanol or aqueous ethanol solutions [73,84].

This approach offers several advantages, including relatively mild operating conditions and compatibility with a wide range of DES compositions. However, it also introduces additional solvent consumption and processing steps, which may partially offset the environmental benefits gained during extraction. Moreover, adsorption efficiency can be influenced by DES composition, water content, and the presence of co-extracted matrix components, necessitating careful system-specific optimization. In addition, key operational parameters such as pH, temperature, contact time, and, in continuous systems, flow rate, play a critical role in governing adsorption and desorption efficiency. These variables are not consistently reported across studies, which limits reproducibility and direct comparison of results.

### 5.2. Liquid–Liquid Extraction and Antisolvent-Induced Precipitation

Liquid–liquid extraction has been less frequently applied to IL- and DES-based extracts, largely due to the strong solvation of polyphenols within the solvent phase, which often results in unfavorable partition coefficients [77]. This limitation is particularly evident for highly polar or strongly hydrogen-bonded phenolics, for which conventional immiscible solvents fail to achieve efficient separation.

Antisolvent-induced precipitation represents a comparatively simple and scalable alternative. By introducing water or ethanol, the hydrogen-bonding network of the solvent system can be disrupted, leading to selective precipitation of poorly soluble polyphenols. This strategy has been successfully applied, for example, to the recovery of ellagic acid from DES extracts, where controlled water addition enabled effective compound isolation while allowing partial solvent reuse [77].

Despite its simplicity, antisolvent precipitation is inherently selective and cannot be universally applied. Highly water-soluble phenolics may remain dissolved, and excessive dilution can compromise solvent recyclability. As such, this approach is most effective when aligned with specific target compounds and solvent compositions.

### 5.3. Integrated Purification Strategies and Solvent Reuse

To address the limitations of single-step purification approaches, several studies have explored integrated strategies that combine extraction and purification within a unified workflow. Examples include DES-assisted solid-phase extraction systems and DES-modified adsorbents, which exploit solvent–solute interactions to enhance selectivity while minimizing solvent switching [47].

Solvent reuse and recycling remain central considerations in these integrated approaches. While complete solvent recovery is challenging due to low volatility, partial recycling of DESs has been demonstrated in multiple systems with limited loss of extraction efficiency over successive cycles [73,84]. However, solvent degradation, accumulation of impurities, and changes in water content can progressively alter solvent properties, highlighting the need for monitoring and regeneration protocols.

### 5.4. Toward Application-Oriented Processing Concepts

An alternative strategy to mitigate downstream challenges involves bypassing solvent removal altogether. Given that many DES components are biocompatible and composed of naturally occurring metabolites, several studies propose the direct use of DES extracts in end-use formulations, particularly in cosmetic, pharmaceutical, and nutraceutical applications [47,60,64]. In such cases, the solvent becomes an integral part of the final product rather than a processing aid to be removed.

While promising, this approach requires careful evaluation of extract stability, bioavailability, and regulatory compliance. Viscosity, solvent–solute interactions, and potential synergistic or antagonistic effects must be thoroughly assessed to ensure product performance and safety.

### 5.5. Implications for Sustainability and Process Design

Collectively, the studies reviewed demonstrate that downstream processing is not a peripheral issue but a decisive factor in determining the true sustainability of IL- and DES-based extraction processes. As highlighted throughout Section 3 and Section 4, solvents that maximize extraction efficiency often present the greatest challenges in terms of purification and recovery. From this perspective, the classification of these systems as “green” cannot rely solely on extraction performance, but requires a comprehensive evaluation of the entire process. In this context, life cycle assessment (LCA) becomes essential, particularly since additional purification steps and solvent recovery operations may offset the advantages observed at the extraction stage.

Future progress in this field will therefore depend on integrated solvent–process design, in which extraction efficiency, solvent recyclability, purification strategy, and end-use application are considered simultaneously. Within this framework, DESs offer particular advantages due to their compositional flexibility, which enables tuning not only for extraction performance but also for downstream compatibility. However, without such holistic design approaches, the practical implementation of smart solvents for polyphenol extraction will remain limited to laboratory-scale demonstrations.

## 6. Critical Overview and Future Perspectives

The increasing interest in ILs and DESs for polyphenol extraction reflects the ongoing search for alternatives to conventional organic solvents. While both solvent families have demonstrated enhanced extraction performance across a variety of plant matrices, a critical analysis of the literature (Table 1, Table 2 and Table 3) reveals that these improvements are often reported without a comprehensive evaluation of solvent recovery, recyclability, and overall process sustainability. Direct quantitative comparison between studies remains challenging due to differences in plant matrices, target compounds, extraction conditions, and reporting methodologies, which limits the ability to establish universal performance benchmarks. Their polarity and hydrogen-bonding capacity, together with their ability to promote specific solvent–solute interactions, can contribute to improved extraction efficiency under controlled conditions, particularly when combined with process intensification strategies such as ultrasound- and microwave-assisted extraction. However, these effects are highly dependent on the specific solvent system and extraction conditions.

Despite these promising results, several constraints have been reported. From a physicochemical standpoint, high viscosity remains one of the most significant limitations, particularly for DESs, as it can hinder mass transfer and reduce extraction kinetics. Although dilution with water and optimization of operational parameters may partially mitigate this effect, such modifications can influence solvent structure and, consequently, extraction selectivity. In addition, while ILs offer remarkable structural tunability, concerns related to toxicity, biodegradability, and environmental persistence have been reported, especially when compared with widely accepted green solvent systems such as ethanol:water mixtures.

Beyond molecular and physicochemical considerations, the translation of laboratory-scale findings into practical applications remains insufficiently explored. Although numerous studies report improved extraction yields, as evidenced in Table 1, Table 2 and Table 3, solvent recovery and recyclability are only sporadically reported, despite being critical parameters for assessing process sustainability. Furthermore, downstream processing steps, including compound isolation and solvent recovery, may involve additional operations or auxiliary solvents that can offset the perceived environmental benefits of IL- and DES-based systems. The development of efficient recovery and reuse strategies is therefore essential to ensure that the reduced volatility and design flexibility of these solvents are not offset by downstream processing challenges. Moreover, variability in experimental conditions across studies hampers direct performance comparison, underscoring the need for greater methodological harmonization to enable reliable cross-study assessment.

A key aspect emerging from the analysis presented in Section 3, Section 4 and Section 5 is that extraction yield should not be considered the sole parameter for evaluating sustainability. A comprehensive assessment must also account for solvent toxicity, preparation complexity, economic feasibility, recyclability, and compatibility with intended applications in food, nutraceutical, or pharmaceutical contexts. In this context, quantitative green chemistry metrics such as the E-factor, Process Mass Intensity (PMI), and LCA provide a more rigorous framework for evaluating process performance. However, these metrics are still rarely reported in studies involving IL- and DES-based extraction of polyphenols, which limits the ability to perform direct comparisons and critically assess their environmental impact. In this regard, DESs composed of naturally derived components appear particularly promising; however, systematic toxicological evaluation and regulatory clarity remain limited.

Future research should therefore prioritize the rational design of solvent systems based on targeted solvent–polyphenol interactions, alongside strategies to mitigate viscosity-related limitations without compromising extraction performance. Equally important is the establishment of robust and scalable solvent recovery approaches, as well as the adoption of more standardized experimental protocols to facilitate meaningful comparison among studies. Addressing these aspects will be critical for advancing from proof-of-concept demonstrations toward technologically viable and environmentally responsible extraction platforms.

Overall, ILs and DESs represent versatile and promising tools for polyphenol extraction; however, their practical relevance within more sustainable extraction alternatives remains strongly dependent on addressing current limitations related to recovery, scalability, and environmental impact. Nevertheless, their long-term relevance within green extraction strategies will depend on achieving a balanced integration of extraction performance, environmental impact, safety profile, and economic feasibility. A more integrated and multidisciplinary approach, combining molecular understanding with process engineering and sustainability assessment, will be essential to consolidate their role in the development of next-generation extraction technologies for bioactive natural compounds.

## Figures and Tables

**Table 1:** Ionic liquids applied to polyphenol extraction.

Ionic Liquid	Matrix	Main Targets	Extraction Technique	Key Outcome vs. Conventional Solvents	Main Limitations	Recovery/ Recyclability	Ref.
[C_4_mim][BF_4_]	*Camellia sinensis* (tea leaves)	EGCG (**1**), ECG (**2**)	UAE	Higher catechin yield than halide-based ILs	Cost, toxicity concerns	Not reported	[30]
[C_4_mim][B]r	*Galla chinensis*	Condensed tannin (**3**)	UMAE	11.7–22% yield increase; time reduced to 1 min	IL recovery, viscosity	Resin-based	[31,32]
[C_4_mim][Tf_2_N]	*Quercus* sp. (oak galls)	Gallic acid (**4**), tannic acid (**5**)	UAE	Strong enhancement of phenolic acid recovery	Long extraction time; hydrophobic IL	Not reported	[33]
[C_2_mim][TCM]	Matcha tea	Total polyphenols	SLE	Selective enrichment, lower total yield than MeOH	Low specificity; yield variability	Not reported	[34]
[C_n_mim][FeCl_4_] (*n* = 1–5)	Tea leaves	Total polyphenols	SLE	Comparable yield to EtOH with easier separation	Specialized synthesis	Efficient magnetic separation; solvent recovery up to 99.8%	[35]
[PA][EDTA]	*Artemisia argyi*	Total polyphenols	SLE	Higher selectivity than MeOH/EtOH	Limited structural diversity	Not reported	[36]
[PA][Mandelate]	*Artemisia argyi*	Flavones (**6**)	SLE	Selective flavone extraction	Narrow target scope	Not reported	[36]
[P_66614_][Cl/Br]	Black liquor	Total polyphenols	LLE	Up to 94% recovery from effluents	Hydrophobicity; reuse limits	Reused once after extraction	[37]
[C_8_mim][ClO_4_]	Aqueous model systems	Gallic (**4**), vanillic (**7**), syringic (**8**) acids	LLE	Efficient, pH-tunable extraction	Safety and regulatory issues	Recovery achieved via pH modulation of extraction medium	[38]
[C_4_mim][Cl] (additive)	PEG–Na_2_SO_4_ ATPS	Phenolic acids	ATPS	Partition coefficient increase from 11.3 to 29.0	Process complexity	Phase recycling possible within ATPS systems	[39]

**Table 2:** Choline chloride-based deep eutectic solvents applied to polyphenol extraction.

DES Composition (HBA:HBD, Molar Ratio)	Matrix	Main Targets	Extraction Technique	Key Outcome vs. Conventional Solvents	Critical Parameters	Recovery/ Recyclability	Ref.
ChCl:Formic acid (1:1)	Palm bark	Total polyphenols	Reflux extraction	Higher TPC than methanol	Acidic DES; long extraction time	Not reported	[47]
Acetylcholine chloride:Lactic acid (2:1) + 30% H_2_O	Fruits, vegetables, spices	Total flavonoids	Stirred extraction	>70% extraction efficiency	Temperature-sensitive	Not reported	[48]
ChCl:Ethylene glycol (1:4)	Orange peel	Total polyphenols	Solid–liquid extraction	Higher yield than 30% EtOH	Water content critical	Not reported	[49]
ChCl:Ethylene glycol (1:2)	Green tea	Total polyphenols	Solid–liquid extraction	Yield up to 20.1%	Moderate viscosity	Not reported	[50]
ChCl:Lactic acid: butane-1,3-diol:H_2_O (1:5:1:1)	Spruce bark	Total polyphenols	Heated extraction	596 mg GAE/100 g dw	Multicomponent complexity	Not reported	[51]
ChCl:Levulinic acid:N-methyl urea	Citrus peel	PMFs, GOFs	Solid–liquid extraction	High simultaneous recovery	Solvent recovery	Not reported	[52]
ChCl:Glycolic acid:Oxalic acid (1:1.7:0.3)	Raspberry by-products	Polyphenols, anthocyanins (**9**)	Solid–liquid extraction	36.7% yield, high purity	Matrix-dependent	Not reported	[53]
ChCl:Glycerol:Citric acid (0.5:2:0.5)	Blueberries	Anthocyanins (**9**)	Solid–liquid extraction	76% anthocyanin recovery	Selectivity shift	Not reported	[54]
ChCl:Oxalic acid (1:1)	Chestnut shells	Total polyphenols	Maceration	4× higher than MeOH	Strong acidity	Not reported	[55]
ChCl:Citric acid (1:2) + 30% H_2_O	*Lilium davidii*	Gallic acid (**4**), kaempferol (**10**), epicatechin (**11**)	Solid–liquid extraction	Higher yields than EtOH	Water balance	Not reported	[56]
ChCl:Propane-1,2-diol (1:2)	*Buddleja globosa*	Luteolin-7-O-glucoside (**12**)	Solid–liquid extraction	Higher yield than 80% MeOH	Moderate viscosity	Centrifugal partition chromatography	[57]

**Table 3:** Non-choline chloride deep eutectic solvents applied to polyphenol extraction.

DES Composition (HBA:HBD, Molar Ratio)	Matrix	Main Targets	Extraction Technique	Key Outcome vs. Conventional Solvents	Critical Parameters	Recovery/ Recyclability	Ref.
Lactic acid:Glycine (5:1) + H_2_O	Saffron waste	Total polyphenols	Stirred extraction	132 mg GAE/g dw	Acidic medium	Not reported	[60]
Lactic acid:Glycine (3:1)	Hops	Total polyphenols	Solid–liquid extraction	+66% vs. 80% MeOH	Solvent polarity	Not reported	[61]
Lactic acid:Glycine (5:1) + US pretreatment	*Sambucus nigra* flowers	Total polyphenols	UAE + stirring	Significant yield increase	Ultrasound pretreatment	Not reported	[62]
Lactic acid:Glycine:H_2_O (3:1:3)	Medicinal plants	Polyphenols	UAE	Best overall performance	Temperature (80 °C)	Not reported	[63]
Lactic acid:Glucose + H_2_O	Industrial by-products	Polyphenols	UAE	Higher yield than MeOH	Water content	Not reported	[64]
Lactic acid:Sodium citrate (15:1)	*Salvia fruticosa*	Total polyphenols	UAE + stirring	79.9 mg GAE/g dw	High viscosity	Hindered by viscosity	[65]
Lactic acid:Sodium acetate:H_2_O (3:1:4)	Mango peel	Total polyphenols	MAE	56.2 mg GAE/g dw	Microwave power	Not reported	[66]
Glycerol:Glycine:H_2_O (7:1:3)	Olive leaves	Total polyphenols	Heated extraction	+18% vs. MeOH	Temperature control	Not reported	[67]
Malic acid:Fructose:Glycerol (1:1:1)	Olive leaves	Total polyphenols	Nanofluid extraction	Higher yield than EtOH	Nanoparticle addition	Nanoparticles complicate recovery	[68]
Citric acid:Lactic acid (1:4)	Olive leaves	Oleuropein (**13**)	Solid–liquid extraction	+8% vs EtOH	Acidity	Not reported	[69]
Citric acid:Glycine:H_2_O (2:1:1)	Olive leaves	Hydroxytyrosol (**14**)	UAE	4× higher than water	Water ratio	Enzyme pretreatment suggests matrix disruption rather than solvent recovery	[70]
Betaine:Glycerol (1:2) + H_2_O	Olive oil	Polyphenols	UAE	773 µg/g extracted	Phase compatibility	Not reported	[71]
Glycerol:Nicotinamide (5:1)	*Moringa oleifera*	Total polyphenols	Stirred extraction	Comparable to UAE	Limited US effect	Not reported	[72]
Glycerol:Xylitol:Fructose (3:3:3)	Fig leaves	Multiple phenolics	MAE	Highest combined recovery	Solvent complexity	Resin based	[73]
Proline:Oxalic acid (1:1) + H_2_O	*Peumus boldus*	Total polyphenols	UAE	Comparable to MeOH	Acid strength	Not reported	[74]
Glycerol:Sodium propionate (8:1)	Onion waste	Total polyphenols	Heated extraction	112 mg/g dw	Viscosity	Not reported	[75]
Malic acid:Glucose:Glycerol (1:1:1)	Pomegranate peel	Polyphenols	Infrared extraction	Highest TPC via IR	Novel technique	Not reported	[76]

## Data Availability

Data sharing is not applicable to this article.

## Abbreviations

The following abbreviations are used in this manuscript:

ATPS	Aqueous two-phase systems
C_2_MIM	1–Ethyl–3–methylimidazolium
C_4_MIM	1–Butyl–3–methylimidazolium
C3G	Cyanidin–3–O–glucoside
ChCl	Choline chloride
DES	Deep eutectic solvent
dw	Dry weight
ECG	Epicatechin gallate
EGCG	Epigallocatechin gallate
EG	Ethylene glycol
GAE	Gallic acid equivalent
GOFs	Glycosides of flavonoids
HBA	Hydrogen bond acceptor
HBD	Hydrogen bond donor
HRE	Heat-reflux extraction
ILs	Ionic liquids
LCA	Life cycle assessment
LLE	Liquid–liquid extraction
SLE	Solid–liquid extraction
MAE	Microwave-assisted extraction
ME	Maceration extraction
MILs	Magnetic ionic liquids
NaDES	Natural deep eutectic solvents
PA	1–Amino–2–propanol
PEG	Polyethylene glycol
PMFs	Polymethoxylated flavonoids
PMI	Process Mass Intensity
SEM	Scanning electron microscopy
TCM	Tricyanomethanide
Tf2N	Bis(trifluoromethylsulfonyl)imide
TPC	Total Phenolic Content
UAE	Ultrasound-assisted extraction
UMAE	Ultrasound-microwave-assisted irradiation
UPAE	Ultrasonic probe-assisted extraction
